# Distribution of bone thickness in the human mandibular ramus – a CBCT-based study

**DOI:** 10.1186/s13005-020-00228-0

**Published:** 2020-06-08

**Authors:** K. Kronseder, C. Runte, J. Kleinheinz, S. Jung, D. Dirksen

**Affiliations:** 1 Department of Cranio-Maxillofacial Surgery, University Hospital Münster, Waldeyerstraße 30, 48149 Münster, Germany; 2 Department of Prosthetic Dentistry and Biomaterials, University Hospital Münster, Waldeyerstraße 30, 48149 Münster, Germany

**Keywords:** Mandibular ramus, Bone thickness, Anatomical grid, 3D reconstruction, Cluster analysis, Ramus groups

## Abstract

**Background:**

The bone thickness of the human mandibular ramus is an important parameter in mandibular surgeries. The aim of this study was to systematically measure the bicortical bone thickness, the ramus dimensions and the position of the lingula. The measurements were tested on significant correlations to the patients’ parameters.

**Methods:**

Based on CBCT scans 150 rami were reconstructed as 3D polygon surfaces. An anatomical grid was adapted to the ramus surface to mark the bone thickness measurement points and to achieve comparability between the measurements on different mandibles. The bone thickness, ramus height, ramus width and the gonion angle were measured. A cluster analysis was performed with these parameters to identify clinically relevant groups with anatomical similarities.

**Results:**

The median distribution of the bone thickness was calculated and visualized in a pseudo-colour map. The mean ramus height was 44.78 mm, the mean width was 31.31 mm and the mean gonion angle was 124.8°. The average distance from the lingula to the dorsal tangent was 53% of the total width and its distance to the caudal tangent was 65% of the total height. Significant correlations between the bone thickness and the ramus proportions could be identified. Age and sex had no significant influence on the mean bone thickness. The measured rami could be divided into two groups by cluster analysis.

**Conclusion:**

The dimensions of the human mandibular ramus can be determined from 3D reconstructed surface models from CBCT scans. Measurements could be made comparable by applying an anatomically oriented grid. A cluster analysis allowed the differentiation of two groups with different bone thickness distributions and geometries, which can be used for the optimization of osteosynthesis systems and their precision of adaptation to different ramus morphologies.

## Background

A large variability of the ramus morphology can be observed in clinical practice. It is primarily determined by the ramus height, width, gonion angle and the position of the lingula as well as the bone thickness, which all will be examined in more detail in this study.

Knowledge of the bicortical bone thickness of the ramus is required in the use of osteosynthesis systems in order to estimate the necessary screw length [1] and ensure an adequate thickness during placement [2].

In orthognathic surgery, the position of the lingula plays an important role in determining the osteotomy line in sagittal and horizontal mandibular osteotomies [3] and the bone thickness seems to be an indicator for the susceptibility to complications during osteotomies [4].

Various studies have been measuring the bone thickness of the ramus at individual points: Susilo et al. [4] measured the bone thickness of the ramus at one point at the level of the lingula as a guide for performing bilateral sagittal split osteotomy in orthognathic surgery and have put their results in relation to the sex and age of the person examined. Also with regard to bilateral osteotomies, Chrcanovic et al. [5] performed measurements on CBCT scans. Seven measurements of the bone thickness were taken at different heights below the mandibular foramen. To determine the mandibular bone thickness, Fujita et al. [6] measured the cortical bone thickness at four dental oriented points in CT images. The data are intended to facilitate implantological interventions such as bone harvesting. To investigate the application of the lag screw technique according to Eckelt for the treatment of condyle fractures, variable morphologies of the mandibular ramus were shown in the coronal plane by Welk et al. [7].

To our knowledge, there are currently only studies in which bone thickness was measured at a limited number of anatomically prominent points. Consequently, data from systematic measurements of the bone thickness of the entire ramus are missing.

Therefore, the aim of this study was to provide such data and to visualize its mean distribution. The height, width and gonion angle as well as the position of the lingula is to be surveyed. Statistical correlations should be evaluated, and a classification of the rami should be carried out.

## Materials and methods

### Pilot study

**Fig. 1:**
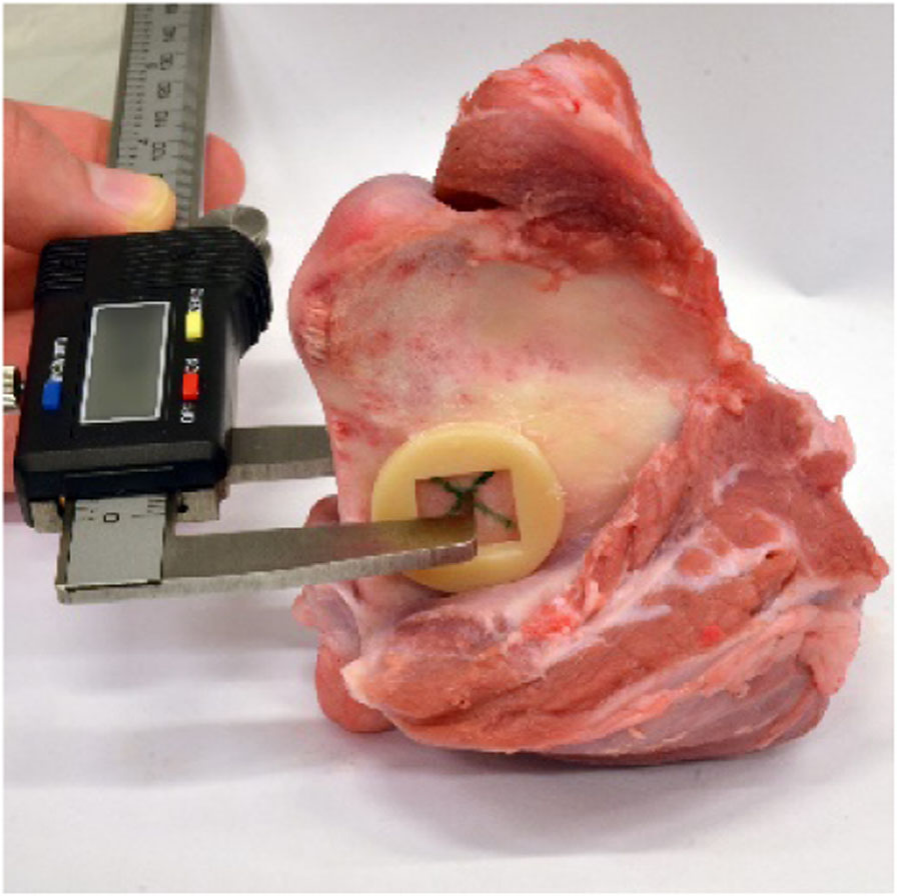
Mechanical thickness measurement on a porcine mandible

**Fig. 2:**
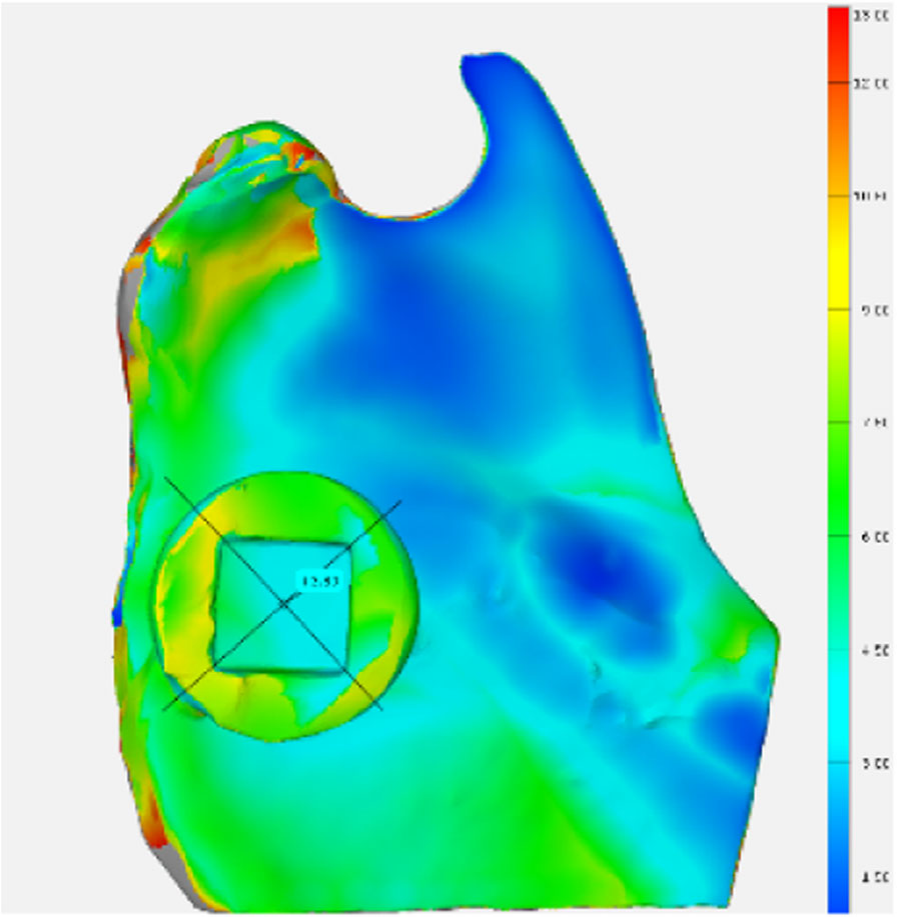
Digital thickness measurement on x-rayed and digitally reconstructed porcine mandible

In a pilot study, the accuracy of the reconstruction and measurement was determined by comparing the applied method to a mechanical measurement. For this purpose, the thickness of five porcine mandibles was measured at a defined position digitally after a CBCT scan and mechanically with a calliper gauge (Figs.1 and 2). The measurements were compared.

### Systematic bone thickness measurements

Scans were included in which the mandibular ramus with muscle and joint process as well as the lower margin of the mandible were completely imaged. Fractures, dysmorphism such as cysts or other structural changes in the bone as well as patients under the age of 18 were excluded from the study. The CBCT scans were made with a Kavo 3D eXam x-ray system (KaVo Dental GmbH, Biberbach, Germany) at a tube voltage of 120 kV and a current of 5 mA and examined with a calibrated diagnostic monitor. The pixel spacing was 0.200/0.200 mm or 0.250/0.250 mm. The total examination time was 14.7 s with an exposure time of 4 s.

From the CBCT scans three-dimensional polygon-models of the mandible jaw were reconstructed by using the medical planning software Mimics (Materialise, Leuven, Belgium) and stored in STL-format. Individual grey scales were identified to differentiate the bone from the soft tissue.

**Fig. 3:**
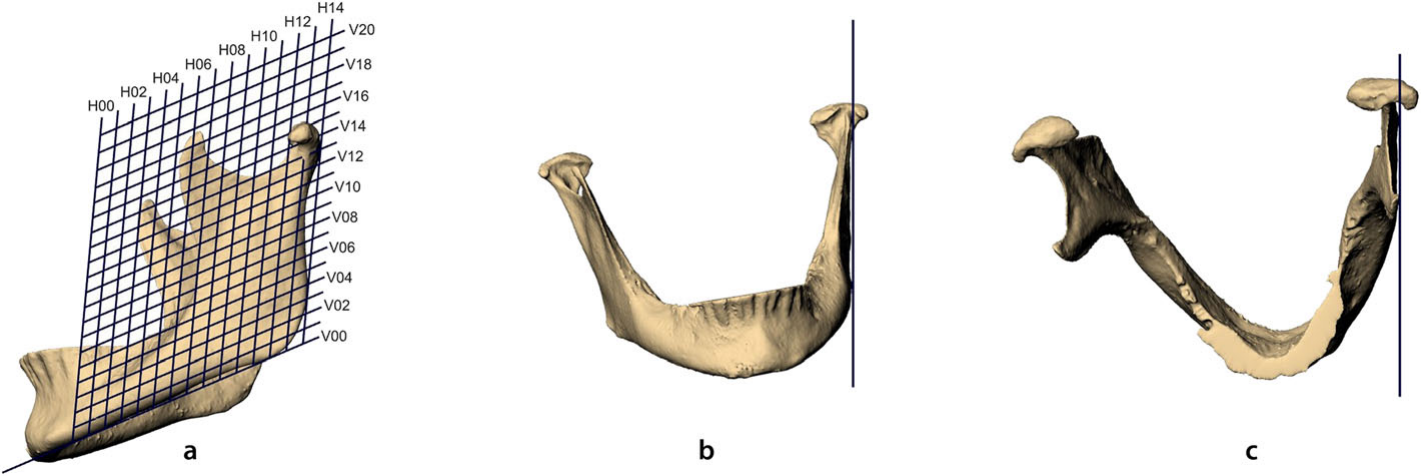
**a**-**c** Adaptation of the grid defining a skew coordinate system in lateral (**a**), frontal (**b**) and cranial (**c**) perspective

The mandibular ramus was defined in this study as that part of the mandible dorsal to the line parallel to the posterior margin and passing through the most dorsal point of the anterior notch by including the muscle and joint process (Fig. 3a).

The measuring points were defined by a two-dimensional grid that was placed on the ramus with the Rhinoceros 6 software (McNeel Europe SL, Barcelona, Spain). The grid was oriented to anatomical landmarks as indicated in Fig. 3a-c: The horizontal and vertical baselines correspond to the caudal and dorsal tangent of the ramus, resp. The grid spacing was adjusted in such a way that the 14th horizontal line is at the level of the sigmoid notch while the 8th vertical line is set to the most dorsal point of the anterior notch, which in a lateral view is the concave retraction between the muscle process and the dental arch. The grid was extended beyond the alignment points to cover the coronoid and articular process. The grid is intended to generate the same number of measuring points in corresponding positions for different sizes and shapes of the rami.

**Fig. 4:**
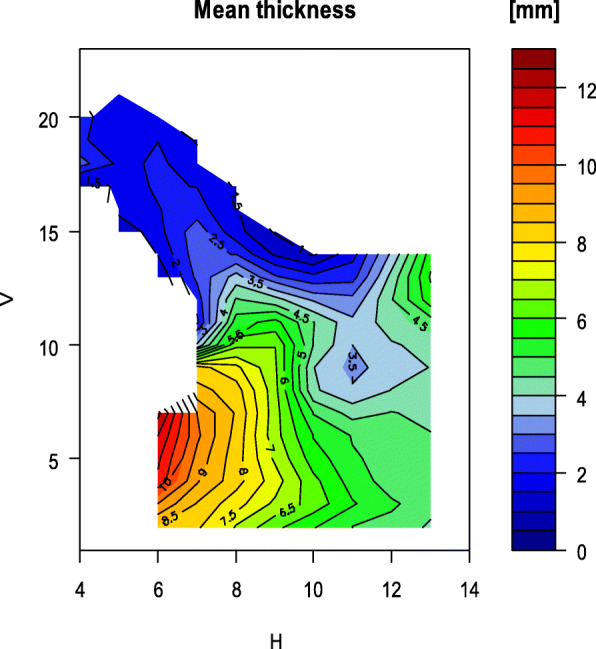
Map of the mean bone thickness of the ramus

**Fig. 5:**
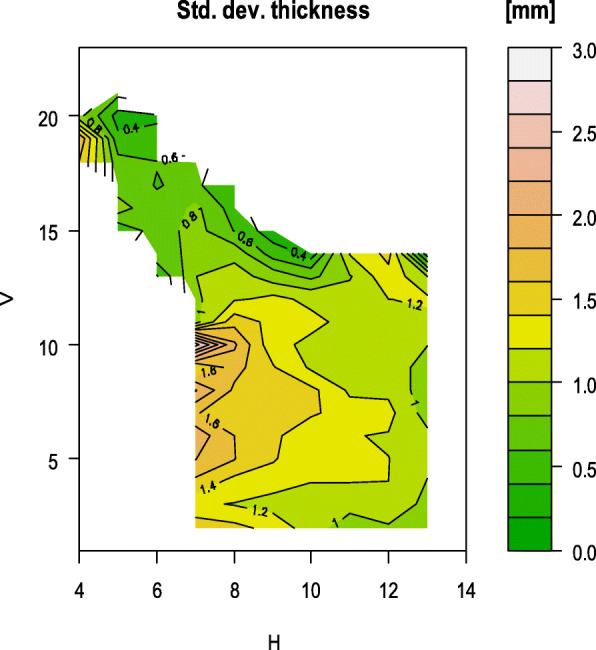
Map of the standard deviation of the ramus

**Fig. 6:**
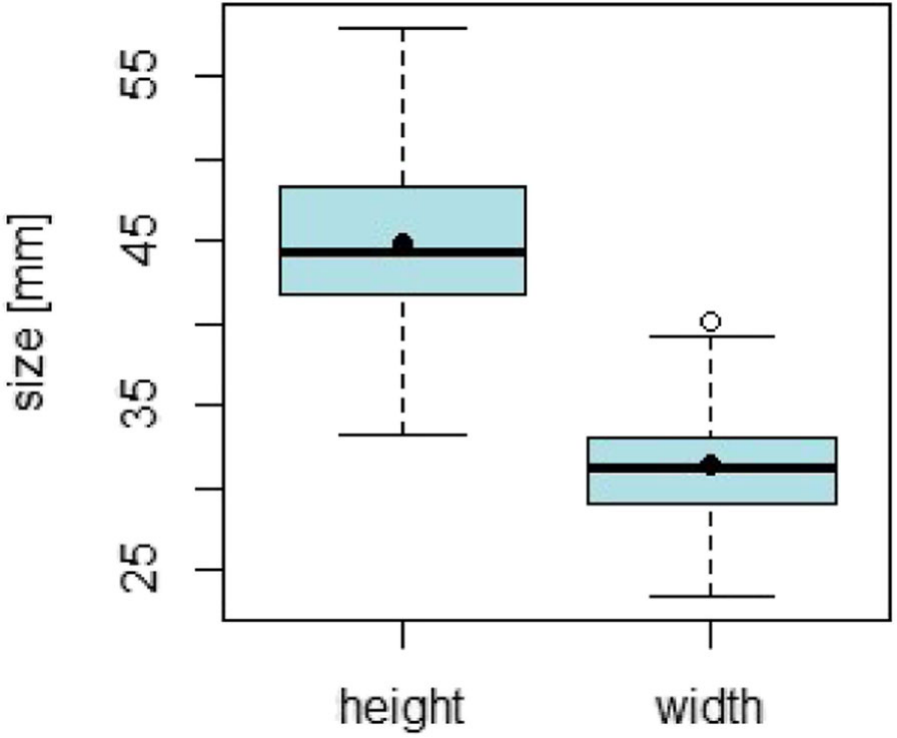
Distribution of ramus height and width; black dots denoting the mean values

**Fig. 7:**
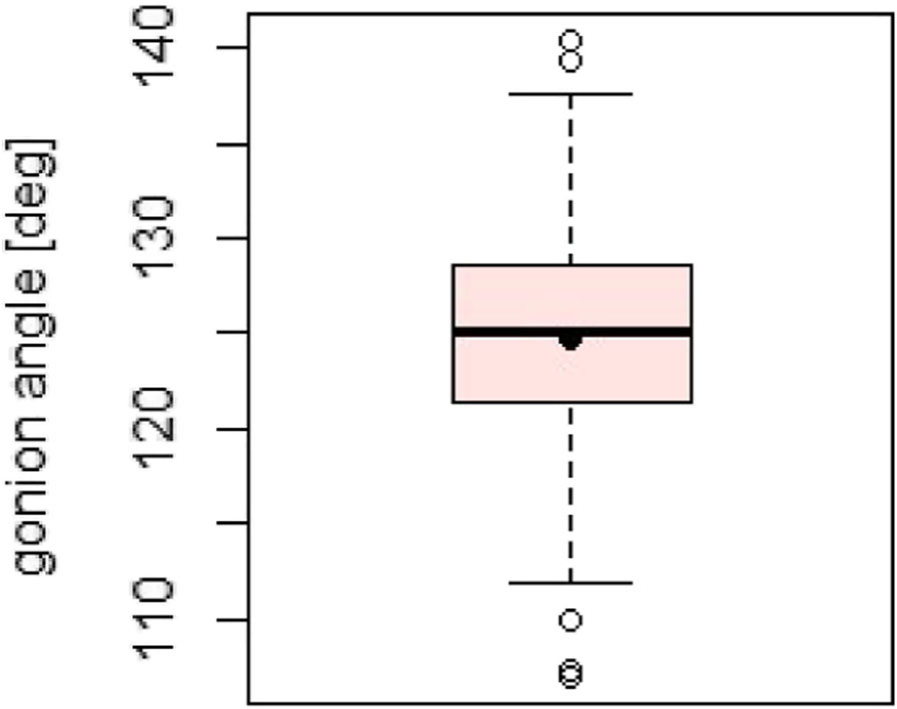
Distribution of the gonion angle; black dot denoting the mean value

**Fig. 8:**
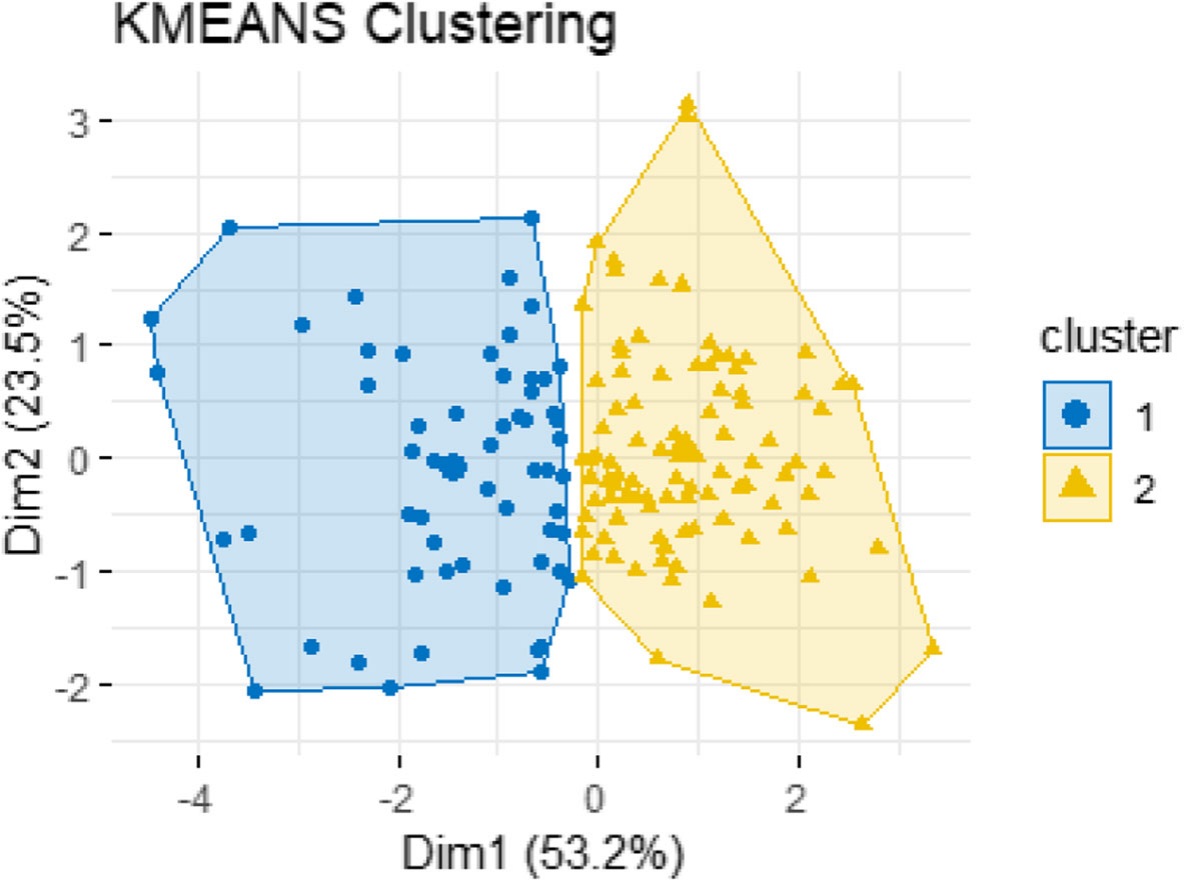
Cluster assignment diagram using two principal components axes

**Fig. 9:**
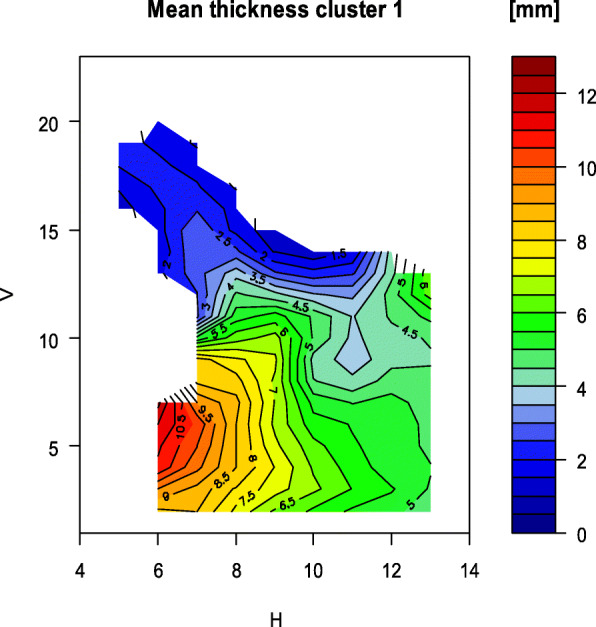
Map of the mean bone thickness of group 1

**Fig. 10:**
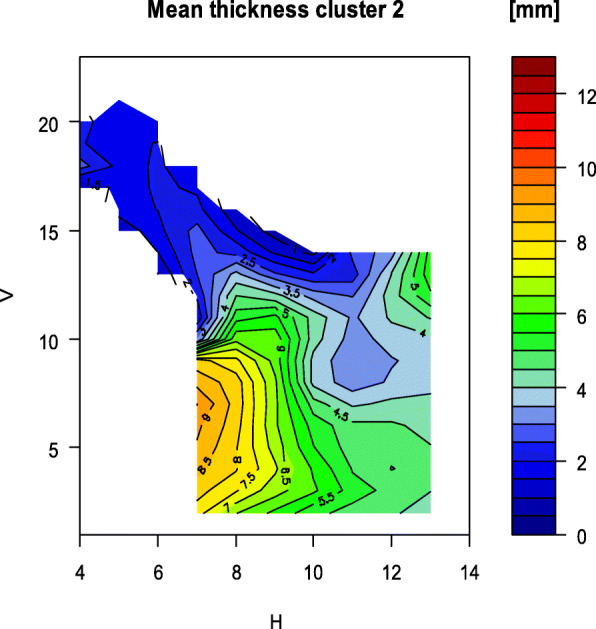
Map of the mean bone thickness of group 2

The intersections of the grid lines marked the measurement points. The measurement of the bone thickness was performed manually by one examiner using the analysis software GOM Inspect (GOM, Braunschweig, Germany). The measurements were taken perpendicular to the surface normal of the ramus at the intersections of the grid lines.

The measured parameters and their descriptions are listed in Table 1.

**Table 1:** Measured parameters

Parameter	Description
Bone thickness	Bone thickness measured at grid intersections
Mean bone thickness	Mean bone thickness referring to all measured points of one ramus
Ramus height	Distance from the horizontal baseline to the sigmoid notch
Ramus width	Distance from the vertical baseline to the anterior notch
Gonion angle	Angle between the vertical and horizontal baseline
Dorsal lingula position	Distance from the tip of the lingula to the dorsal tangent of the ramus
Caudal lingula position	Distance from the tip of the lingula to the caudal tangent of the ramus

Statistical analyses were performed using the software R [8] and RStudio [9].

The k-means method was used for cluster analysis, using the NbClust-library of R.

The statistical significance was set at *p* < 0.05. All analyses were regarded as explorative and *p*-values interpreted descriptively. Therefore, no adjustment for multiple testing was performed.

## Results

### Pilot study

The comparison between the digital and the mechanical measurements showed a mean difference of 0.25 mm (*n* = 5; SD = 0.24 mm). The accuracy is consistent with the accuracy of the CBCT scans, which is limited by the voxel size.

### Systematic bone thickness measurements

Applying the inclusion and exclusion criteria, 150 rami from 81 CBCTs could be selected from a total of 175 scans from the database of the Clinic for Oral and Maxillofacial Surgery Münster. The age ranges from 18 to 88, the mean age is 54.7 (SD = 20.35; 37 males, 44 females).

On average, 81 measurements of the bone thickness could be taken per ramus. For each grid point, the mean value was calculated from all thickness measurements and visualized in false colours. The maps below show the distribution of the mean bone thickness of the mandibular ramus (Fig. 4) and the standard deviation of the measurements (Fig. 5). Rami with their different shapes were incorporated into these graphics, so that the resulting shapes in Fig. 4 and Fig. 5 are summations of different ramus outlines.

In the area bounded by the grid lines H10 to H13 and V7 to V12 where the mandibular foramen is located (Fig. 4), the average bone thickness is 3.5–4 mm. At the sigmoid notch and in the area of the coronoid process, the mean bone thickness is 1–3 mm. At the articular process (H12/V13) the bone thickness increases to an average of 4.5–5 mm.

Following Kolmogorov-Smirnov tests, normal distributions were assumed for the parameters mean bone thickness, height, width, angle and the dorsal and caudal lingula position.

The results of the descriptive statistical analysis are shown in Table 2.

**Table 2:** Measured parameters with mean values, standard deviation and minimum and maximum value

Parameter	Mean Value	SD	Min-Max
Mean bone thickness	4.83 mm	0.73 mm	2.72–7.03 mm
Height	44.78 mm	4.94 mm	33.2–58 mm
Width	31.31 mm	3.28 mm	23.4–40.2 mm
Gonion angle	124.80°	6.01°	107–140.4°
Dorsal lingula position	16.43 mm	1.69 mm	11.8–21.8 mm
Caudal lingula position	28.98 mm	3.89 mm	21.8–43.8 mm

The statistical results of the height, width and angle measurements are shown in the lower graphs as box plots (Figs. 6 and 7).

The average dorsal lingula position (Table 2) corresponds to 53% of the mean ramus width. Its average caudal position is equivalent to 65% of the mean ramus height.

A significant correlation between height and width, width and angle and height and angle is observed. There is a weaker but significant correlation between height and mean bone thickness, width and mean bone thickness and age and angle (Table 3).

**Table 3:** Results of correlation analysis of the geometrical parameters. r denotes the Pearson correlation, CI the 95% confidence interval and p is the local significance value

Parameters	r	CI	p
height – width	0.56	[0.43; 0.66]	< 0.001
width – angle	− 0.43	[− 0.56; − 0.29]	< 0.001
height – angle	− 0.57	[−0.67; − 0.45]	< 0.001
height – mean bone thickness	0.17	[0.01; 0.33]	0.032
width – mean bone thickness	0.25	[0.09; 0.40]	0.002
age – angle	0.31	[0.16; 0.45]	< 0.001
age – height	−0.13	[−0.29; 0.03]	0.11
age – width	−0.22	[−0.37; − 0.06]	0.007
mean bone thickness – age	−0.003	[− 0.16; 0.16]	0.97

No significant correlation between the mean bone thickness and age or sex was found.

According to Welch’s test there is no difference between the left and the right ramus concerning height, width, angle and the position of the lingula.

A k-means cluster analysis was carried out based on the parameters height, width, mean bone thickness and gonion angle. The number of clusters was determined heuristically after evaluating a hierarchical cluster plot.

Accordingly, the rami were divided in two subsets. The mean values of the parameters and standard deviations for each group are listed in Table 4.

**Table 4:** Mean values of height, width, mean bone thickness and gonion angle for the two identified groups

Parameters	Bone thickness (mm)	Height (mm)	Width (mm)	Gonion angle (°)
Group 1	5.16 (SD = 0.78)	49.07 (SD = 3.90)	33.93 (SD = 2.93)	120.60 (SD = 5.42)
Group 2	4.62 (SD = 0.61)	42.15 (SD = 3.45)	29.71 (SD = 2.31)	127.37 SD = 4.80)

The results are visualized by using the first two principal components as axes (Fig. 8).

The rami in group 1 have a larger height and width, a larger mean bone thickness and a smaller gonion angle in comparison to group 2.

In contrast to the standard deviation when all rami are examined, it is smaller for the height, width and the gonion angle within the two groups. The standard deviation of the mean bone thickness, considering all measured values of one ramus, is approximately the same size. However, as the Figs. 9 and 10 illustrate, significant local differences between the individual grid measurements can be identified.

The distribution of the bone thickness for the two groups is shown below (Fig. 9 and Fig. 10). In line with the lower mean bone thickness in group 2, there are more prominent thin areas displayed in the map (Fig. 10). The areas with thin bone on the foramen are more extensive in group 2.

## Discussion

In our study the distribution of the bone thickness of the ramus was examined based on reconstructions from CBCT scans using existing data from the clinic’s database. As the images were taken with a medical indication, a bias had to be contemplated. To address this problem, patients with morphological abnormalities such as fractures, cysts or other pathological lesions of the mandible were excluded.

For each measurement using X-ray images and 3D reconstructions, measurement inaccuracy must be considered. By comparing the applied reconstruction method to mechanical measurements, it could be determined in a pilot study, that the accuracy was in the range of the voxel size of the CBCT scans. Other studies came to the conclusion that CBCT and CT imaging techniques rendered comparable measurement accuracies [10] and have been verified by in-situ measurements [11].

By applying an anatomical grid, comparable thickness measurements could be achieved for an average of 81 points per ramus for which the mean bone thickness could be calculated. This allowed for a more detailed inspection of the ramus anatomy than in previous studies, where the bone thickness of the mandibular ramus was measured at individual points for specific questions, for example to analyse the bone thickness in the course of sagittal split osteotomies by taking one thickness measurement [4] or seven bone thickness measurements at different levels of the ramus [5] or to evaluate the bone thickness for implantation and bone harvesting as performed by Chrcanovic et al. [6], where four measurements were taken.

Of course, individual mandibles presented a deviation from the calculated mean values, which was found in the standard deviation. Nevertheless, the acquired data can serve as a guide for identifying areas with adequate bone thickness for placing screws.

Our results showed that the patients’ age has no influence on the mean bone thickness of the ramus, which confirms the results of Chrcanovic et al. [5] and Susilo et al. [4]

Zhou et al. showed that there is no significant difference in the gonion angle between men and women [12], which is consistent with our data. According to that study „the mean mandibular angle was 125.1° in males and 124.1° in females “[12]. Our evaluations show comparable results with an angle of 124.93° for females and 124.64° for males. However, the authors of a study on the measurement of panoramic images stated, that the gonion angle is smaller in men than in women. Since the selection criteria and sample size of that study are similar to ours, the different imaging methods should be considered as the reason for the discrepancy [13].

Susilo et al. [4], Chrcanovic et al. [5] and Scomparin et al. [16] found no significant sex difference in the mandibular bone thickness, which corresponds with our results.

Moreover, we could not detect any significant difference in ramus height and width between men and women. In contrast, Indira et al. and Saini et al. proposed the mandibular height and width as a means of determination of sex, as they detected a greater height and width in males than in females [14, 15].

Our study showed a small but significant correlation between the patient age and the gonion angle, which is in contradiction to the results of Abu-Taleb et al., who found no such correlation [13]. Abu-Taleb et al. used the same anatomical landmarks for angle measurement, but these were measured on panoramic images.

When comparing these results with the literature, it should be considered that the measurements may have been based on different ethnic groups. Further studies should be carried out to identify possible differences.

The measured rami could be assigned to two groups by cluster analysis based on the parameters of bone thickness, height, width and gonion angle. Similarities and differences in the distribution of bone thickness are shown in the maps, which were based on the measured thicknesses on the anatomical grid. The grouping of rami with similar parameters results in a lower standard deviation within the formed groups. This allows for more precise statements, for example on the distribution of bone thickness over anatomically related rami.

## Conclusion

In our study the proportions of the mandibular ramus and the position of the lingula as well as the bone thickness could be measured with an accuracy in the range of the voxel size of the CBCT scan.

By applying a skew-angled grid oriented to anatomical landmarks, the measurements could be made comparable and visualized with the help of false-colour maps. Statistical evaluations revealed significant correlations between height and width, width and angle and between height and angle. No significant correlation between the mean bone thickness and age or sex was found.

Further investigations have to be carried out for hypothesis testing.

In addition, a cluster analysis was performed to group the measured rami according to anatomical similarities.

In clinical practice, these results can support the selection of anatomically appropriate osteosynthesis materials and provide guidance for screw positioning. In addition, the two distributions of mean bone thickness identified by the cluster analysis enable the specification of two sets of osteosynthesis plates for the ramus with insertion slots in the areas with higher bone thickness.

Taking into consideration a broad age spectrum, general conclusions could be drawn about the ramus morphology, which can be useful for fracture treatment or orthognathic surgery, among other things. In order to collect data specifically for the target group of orthognathic patients, further studies with a more specific age selection should be aimed for.

## Data Availability

The data sets examined for this study are available from the corresponding author upon reasonable request.
